# The role of Research‐Practice Ambassadors in strengthening socially just and equitable partnership processes

**DOI:** 10.1002/ajcp.70047

**Published:** 2026-01-25

**Authors:** Danielle R. Hatchimonji, Lauren McNeela, Zumana Noor, Melissa Stoffers, Tia N. Barnes, Kira Branch, Danika Perry, Amanda Parks, Mariam Berthe

**Affiliations:** ^1^ Nemours Children's Health Wilmington Delaware USA; ^2^ Thomas Jefferson University Philadelphia Pennsylvania USA; ^3^ Ann & Robert H. Lurie Children's Hospital of Chicago Chicago Illinois USA; ^4^ Feinberg School of Medicine Northwestern University Evanston Illinois USA; ^5^ University of Delaware Newark Delaware USA; ^6^ University of Nevada Las Vegas Nevada USA

**Keywords:** partnership processes, racial equity in education, Research‐Practice Partnerships, school‐based intervention research, school‐based research methods, social justice, socially just research

## Abstract

Research‐Practice Partnerships seek to close the research‐practice gap through developing collaborative, authentic partnerships between researchers and community members. Our team has leveraged Research‐Practice Ambassadors to support socially just and equitable partnership processes in schools. We describe how the Ambassadors can support these types of long‐term partnerships by building trusting relationships, uplifting practitioner voice and lived experiences, and strengthening bidirectional communication across research and practice teams. We provide details about the inputs and activities for the Ambassador role, the potential benefits of this role in strengthening partnership processes, and case examples of how our team has used the Ambassador role to strengthen partnerships. Future directions of this work should evaluate the impact of using this Ambassador role and incorporate critical analyses of power.

## Highlights

1. We describe a research methodology to support research‐practice partnerships in school‐based intervention research: Research–Practice Ambassadors.
2. The Research–Practice Ambassador methodology builds from principles of community psychology to enhance relationships and bidirectional communication between the research and practice teams.
3. Future research should critically examine power and test the theorized pathways through which Research–Practice Ambassadors can support socially just and equitable partnership processes.

Historically, there is a well‐documented disconnect between research findings and the successful implementation of these findings through interventions or programs (e.g., Quintana & Mahgoub, 2016). Previously, this disconnect was attributed to barriers with dissemination of findings or motivating others to enact the findings; however, recent papers have put forth the idea that the observed disparity is better understood as a bidirectional problem (e.g., Farley‐Ripple et al., 2018). Specifically, the gap between the proposed research outcome and observable outcomes in practice is likely due to an inability of information to flow freely and equitably between research groups and practitioners.

The bidirectional nature of the research‐practice gap suggests a more collaborative approach may reduce this gap, and the emergence and increased popularity of research‐practice partnerships (RPPs) is thought to offer one such approach. RPPs can be defined as “long‐term, mutually‐beneficial collaborations geared toward identifying problems of practice and testing solutions for improvement” (p. 1, Grant et al., 2023). Because of this collaborative structure aimed at mutuality, RPPs are well‐positioned to advance social justice, equity, and antiracist efforts (Henrick et al., 2019). In other words, RPPs have the potential to address problems of practice that are related to injustice or inequities by centering social justice as not only an outcome but as a consistent value of the partnership.

In this paper, we describe how we have used the role of a Research‐Practice Ambassador to support socially just and equitable partnership processes. Social justice aims to advance equity, uplift diverse perspectives, disrupt oppression, critically examine and challenge power differentials, and dismantle racist practices so that all members of society have access and can benefit from these spaces (Bell, 2007; Vetter et al., 2022). Broadly speaking, our partnered research works with educators to advance equity and social justice, with the ultimate goal of supporting the wellbeing of all staff and students. The Ambassadors primarily support partnership relationships and communication by building trust and authentic relationships with staff, providing links between the research and practice communities, shifting power dynamics by uplifting practitioner and student voice, and strengthening bidirectional communication. These activities are thought to support both equity and efficacy in the RPP, while aiming to develop a meaningful and sustainable partnership between schools and researchers.

The purpose of this paper is to describe the Ambassador role and delineate the proposed model for how this role can be a tool to support socially just partnership processes in schools. We draw on our experiences in four school districts to provide brief case examples. This paper provides (1) a template from which others may build a similar Ambassador program and (2) a framework that can be used to test the proposed impact of the Research Practice Ambassador on socially just partnership processes.

## SOCIALLY JUST AND EQUITABLE PARTNERSHIP PROCESSES

RPPs have been developed in education settings to center the relationship between research groups and community/practice groups to collaborate across all research steps (Vetter et al., 2022). The recently updated definition of RPPs provided by Farrell et al. (2021) centers mutuality with equitable transformation as the ultimate goal: “A long‐term collaboration aimed at educational improvement or equitable transformation through engagement with research. These partnerships are intentionally organized to connect diverse forms of expertise and shift power relations in the research endeavor to ensure that all partners have a say in the joint work” (p. 5). To shift these power relations, RPPs offer a model in which participants are considered co‐researchers, with expert knowledge of the systems in which they are involved (Levac et al., 2019). This approach helps to amplify voices that have been historically undervalued or neglected during the research process. The collaborative nature of this research model aims to close the gap between research and practice through collaboration in project design, delivery, and overall agenda.

RPPs and similar forms of participatory and engaged research have been an increasing area of inquiry over the past three decades, culminating in an empirically‐derived conceptual model that describes the components of this work (Ortiz et al., 2020). As presented by Kastelic et al. (2018), community‐engaged and participatory research includes four domains: (1) contexts, (2) partnership processes, (3) intervention and research, and (4) outcomes. When RPPs successfully center social justice and equity throughout these domains, they have the potential to meaningfully advance social justice (Farrell et al., 2021; Henrick et al., 2019; Ortiz et al., 2020).

Unfortunately, developing sustainable and equitable research partnerships can be challenging, especially when considering partnership processes. For example, in a review of RPP psychology studies, it was noted that projects rarely, if ever, involved community members across all stages of research (Levac et al., 2019), which may hinder the maintenance of a true collaborative partnership. Teemant et al. (2021) note that a primary challenge in developing shared perspectives in school partnerships is a lack of social interaction across educators, families, and community members. Furthermore, Vetter et al. (2022) conducted a literature review of how RPPs in education relate to equity and justice. They defined equity‐centered RPPs as purposefully addressing issues of power and privilege within communities, within the structure of the partnerships themselves, and within the problems of practice (Vetter et al., 2022). These partnerships are grounded in equity and justice frameworks, clearly define key terms, use equity‐aligned research methods, and advance equitable outcomes and systems (Vetter et al., 2022). They found that only 9% of the partnerships explicitly centered equity and justice, while the remaining partnerships were only indirectly related to equity, without a clear framework. Specifically, many failed to define equity‐related goals, did not use equity‐centered theoretical frameworks, used vague equity language, did not address underlying systemic inequities, and most did not center marginalized voices in their partnerships (Vetter et al., 2022). The authors of these reviews indicate that one barrier may be entrenched power dynamics that impede RPPs in successfully developing socially just and equitable processes.

Imbalances in power among researchers, practitioners, students, and families can hinder open communication and collaborative decision‐making (Duclos et al., 2024; Penuel et al., 2015). Indeed, practitioners in RPPs often experience ramifications of current and historical power differentials such that their professional opinions are disregarded when collaborating on research protocols (Burk et al., 2018). When power differentials are not addressed, it is unlikely that all partnership members will define problems of practice and thereby develop research questions that guide the partnership's work. Thus, without a continued focus on equitable partnership processes, an RPP can unintentionally maintain an imbalance of power and continue to marginalize underrepresented voices (Vetter et al., 2022).

As research on equitable partnerships continues to advance, there are some clear recommendations to enhance socially just partnership processes and address these power differentials. First, it is important to prioritize the partnership relationships over the research agenda, with a focus on building trust (Coburn et al., 2013; Rosenquist et al., 2015). This emphasis on relationships is challenging because it requires significant investment of time, which can often be a barrier to RPPs in which research incentives require urgency and academic deliverables (Levac et al., 2019). Successful prioritization of relationships means a flattening of power hierarchies that supports developing a shared understanding of partnership goals (Teemant et al., 2021). In support of trusting relationships and shared goals, it is essential to maintain open communication and transparency across all those involved in the partnership process (Coburn et al., 2013). This means prioritizing bidirectional communication between the research and practice teams, rather than the typical flow of information from researchers to practitioners. Again, the investment of time this bidirectional flow of information requires is a significant barrier both for practitioners in schools who are overburdened with their own deliverables and for researchers with time‐sensitive academic promotion incentives.

Finally, an additional strategy to support socially just and equitable partnership processes is to use specific research methods that can strengthen both relationships and bidirectional flow of information. As Vetter et al. (2022) highlight, exemplar equity‐focused RPPs used research methods that supported examination of power differences (e.g., Denner et al., 2019) and that amplified the voices of students or practitioners (e.g., Bevan et al., 2017). Taken together, the current literature suggests that although actualizing equitable and socially just partnership processes is challenging, time‐consuming, and fraught with entrenched power differentials, it is possible to overcome these challenges by prioritizing relationships, bidirectional communication, and equity‐focused research methods.

## THE AMBASSADOR ROLE AS A SUPPORT TO SOCIALLY JUST AND EQUITABLE PARTNERSHIP PROCESSES

**Figure 1:**
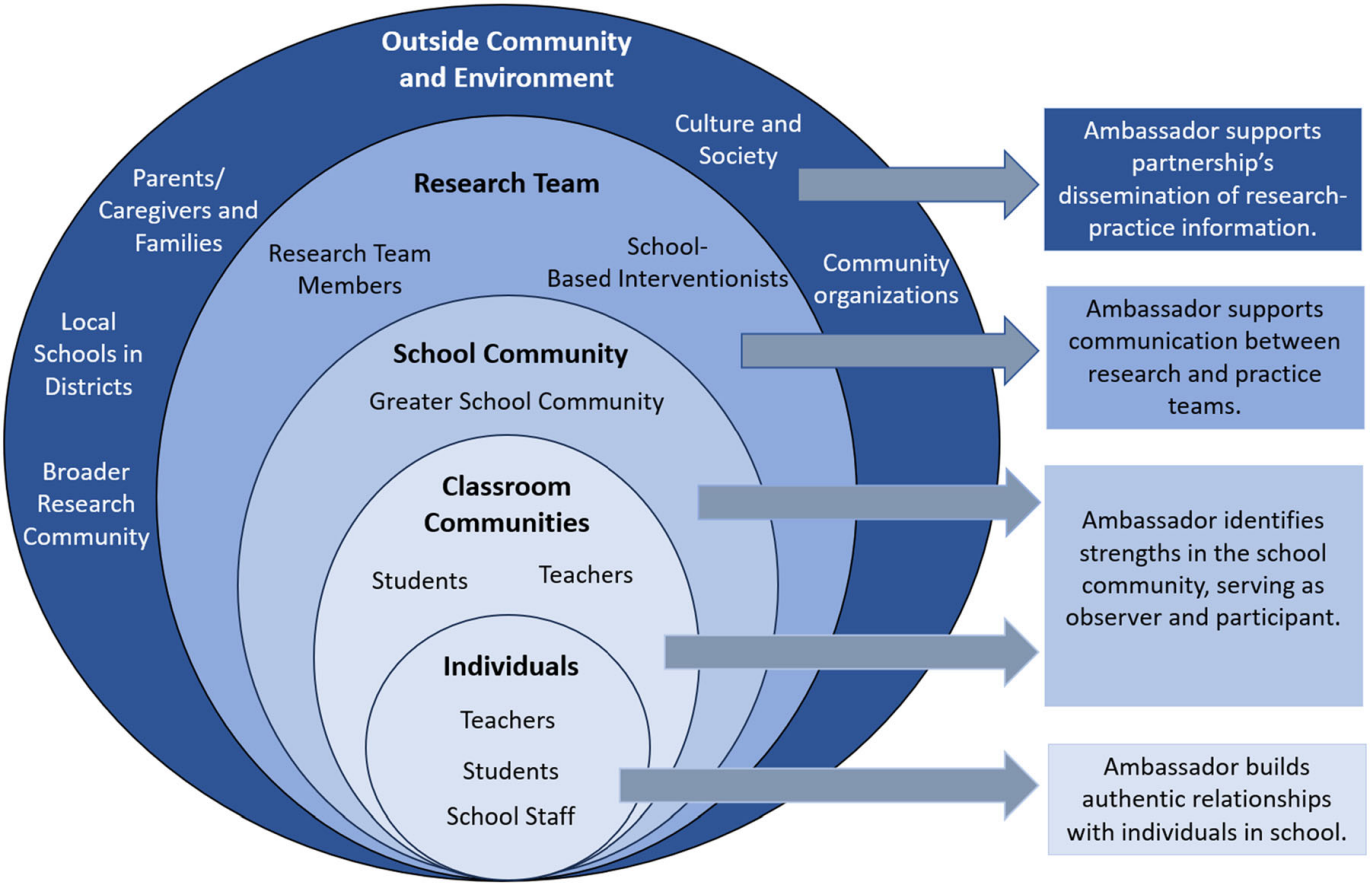
Socioecological model of the Research‐Practice Ambassador impact on Partnership Processes.

We have used the Research Practice Ambassador role to address this need to support socially just and equitable partnership processes. The model we describe here was built upon the work of community psychologist Maurice Elias and colleagues who formed RPPs in schools throughout New Jersey over four decades (Linsky & Elias, 2018). As used by our team, the Research‐Practice Ambassador leverages ethnographic techniques to facilitate a deeper understanding of the multiple levels of the socioecological context in a school environment (Figure 1). We acknowledge that, like most research tools, the Ambassador role does not inherently support socially just and equitable partnership processes. Yet, when used to support relationships and communication that disrupts power differentials, the Ambassador role can be one important component of socially just and equitable partnership processes.

## AMBASSADOR AS OBSERVER AND PARTICIPANT IN THE SCHOOL

The Ambassador role builds on previously described methodologies, such as critical ethnography and practices embedded in a community psychology approach to research. Case and colleagues (2014) describe how “ethnography with the goal of social action through collaboration” is well‐aligned with the values of community psychology. In fact, community psychologists often use participatory research methods to amplify voices of marginalized groups and advance social justice and community wellness (Levac et al., 2019).

The Ambassador role shares characteristics with the “participant observer” and “observer participant” methodologies used in organizational ethnography (Mears, 2013; Moeran, 2009). The goal of these methodologies is to develop a deep understanding of social phenomena; for example, understanding the economics and politics of the modeling industry (Mears, 2011) or substance abuse among transnational Mexican farmworkers (García, 2015). The “observer” and “participant” approaches to understanding social phenomena represent two ends of a spectrum. In the school context, an observer, who is a “fly on the wall,” remains an outsider and takes on no active engagement in day‐to‐day activities. The participant, on the other hand, is an active member of the school who takes part in the social environment, either as a staff member or student. The participant role offers opportunities for a deeper understanding of social phenomena by offering access to the “backstage” context (Moeran, 2009), while the observer role offers a distance that can provide insights not visible to the school community members (Mears, 2013).

Important to socially just partnership processes, “critical ethnography” calls for attention to positionality, power and control, and disruption of the status quo (Hammoudi & Borneman, 2009). Relatedly, a collaborative, participatory ethnography, as described by Lassiter (2005), emphasizes the same level of community partnership and engagement as community‐based participatory research. The RP Ambassador role balances the “observer” and “participant” roles and leverages these elements of criticality and partnership from ethnographic methods. In this way, the Ambassador contributes to a deeper understanding of the school context than is typically available to research teams in schools.

As the Ambassador attempts to strike a balance between the “participant” and “observer” roles while building authentic relationships within the school community, the Ambassador also takes on a liaison role to support information sharing. This combination of understanding the social context (ethnographic methods) and the bidirectional flow of communication (liaison) supports RPPs in shared governance of the partnership. Thus, the Ambassador can help the broader partnership examine power dynamics inherent in the conventional ways of partnering and support naming, reflecting, and transforming inequitable processes (Teemant et al., 2014). In this way, Ambassadors can uplift practitioner voice in support of shared agreements for research priorities and data ownership policies that meet the needs of all partnership members.

**Figure 2:**
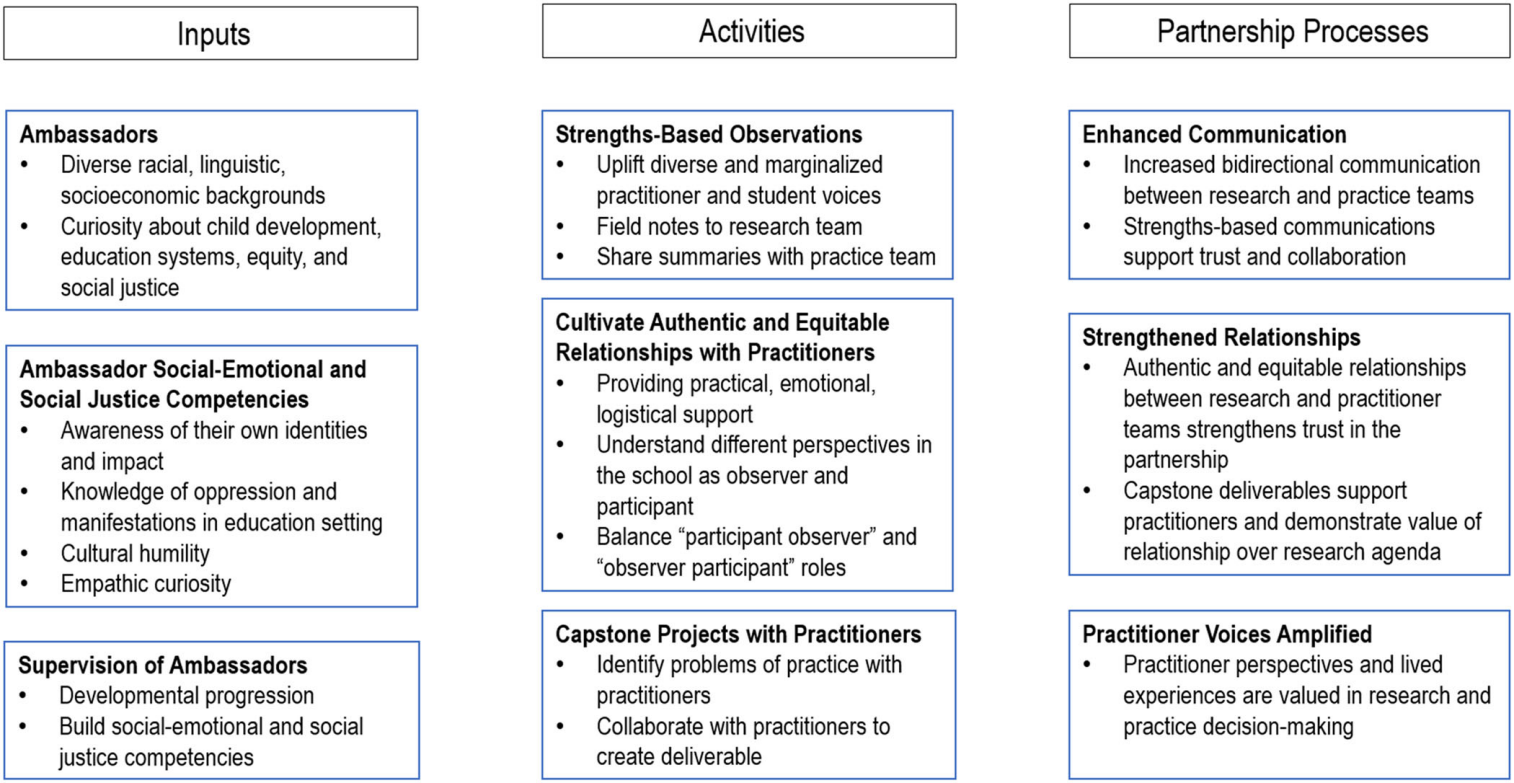
Logic model depicting the impact of the Research‐Practice Ambassador on partnership processes.

In addition to ethnographic and liaison roles, Ambassadors take on a participatory action research role to respond to needs they identify. As they identify and respond to needs in partnership with school practitioners, the Ambassador further builds trusting relationships. Thus, the Ambassador role is positioned to support bidirectional communication, trusting relationships, and more equitable power in the partnership processes at every stage of research, from conceptualizing to conducting, interpreting, and disseminating. Toward these goals, our team has engaged Ambassadors in three key activities: (1) sharing strengths‐based observations, (2) cultivating authentic and equitable relationships, and (3) developing collaborative capstone projects. Through intentional engagement in these activities, we propose that Ambassadors' consistent (e.g., weekly) involvement in a school system over several years can support socially just and equitable partnership processes in a RPP (Figure 2).

## INPUTS TO EMBED RESEARCH‐PRACTICE AMBASSADORS IN PARTNERSHIP PROCESSES

### Selecting ambassadors

Advanced undergraduates or junior graduate students benefit from this Ambassador position because they have the maturity to handle the ambiguity inherent in this role, and at the same time, their curiosity about the school system is a powerful guiding force for their work. The role is flexible to accommodate different professional interests, though most students who take on this role have an interest in a career as a helping professional. Intentionally recruiting a group of Ambassadors from diverse racial, linguistic, and cultural backgrounds supports a diversity of perspectives in the RPP team. From the outset, Ambassadors should demonstrate both curiosity and humility about child development, some knowledge of the local community, and passion for the pursuit of social justice. We recommend Ambassadors be compensated for their work through grant funding, University work study funds, or course credit.

### Supporting and supervising ambassadors

To support Ambassadors' capacity for strengths‐based observations and cultivating trusting relationships, for every 6 h an Ambassador spends in a school, they receive at least 1 h of live group supervision with research‐practice staff. Supervisors must have knowledge and awareness of the specific research and practice contexts and be well‐versed in and committed to focused efforts on social justice and equitable transformation. The supervisor must also understand ethnographic methods, community psychology principles, and content‐specific knowledge related to the broad research questions at hand. Supervisors also provide support for Ambassadors to prioritize their own mental health and wellbeing, with particular attention to how the Ambassadors' histories of marginalization and trauma may influence their experience of the Ambassador role.

### Training ambassadors

Ambassadors must use social‐emotional and social justice competencies to foster authentic relationships within the school and make strengths‐based observations of the school's social environment. Social justice competencies refer to the social‐emotional awareness, knowledge, and skills to interact effectively with diverse people in diverse contexts and foster equity and inclusion (Goodman, 2013). At the outset of their roles, Ambassadors may differ in their skills in each of these areas, but when they demonstrate cultural humility, empathic curiosity, and a willingness to learn, they can develop these skills in the field through intentional supervised experiences (Flores et al., 2014).

The Ambassador supervision process follows a developmental progression, beginning with highly structured exercises to build observational, social‐emotional, and social justice competencies. During the foundational training period before they enter the school system, Ambassadors read research and practice articles relevant to the broad research questions. For example, in our research, these foundational readings would include an overview of current and historical inequities (e.g., manifestations of racism in the school setting) as well as strengths and protective factors (e.g., school belonging, family racial socialization). As Ambassadors prepare to enter the school environment, supervision topics focus on their ability to balance the participant and observer roles, including an awareness of their own sociocultural identities, their biases, power dynamics in relationships they seek to build, and the impact of their presence in different spaces in the school. As Ambassadors have their first few school visits, supervisors facilitate discussions about the impact of intersecting identities on perspectives and experiences to support the Ambassadors' development of critical consciousness in the school context. Over the course of a school year, as Ambassadors develop their own observational skills and relationships in the school, supervision topics and related articles shift to understanding the specific problems of practice identified by the Ambassador in collaboration with students or practitioners. If challenges arise, supervisors support the Ambassadors in leveraging or building upon their own social‐emotional skills, like social awareness and self‐awareness, to continually identify the core values and priorities of different members of the school community.

## CASE EXAMPLES OF RPP AMBASSADOR ACTIVITIES AND OUTCOMES

Beginning in 2023, we have applied the RPP Ambassador model to a specific line of school‐based intervention research, Actions Against Racism. This project is a school‐based intervention that aims to promote student and staff wellbeing by supporting five actions against racism: talking, recognizing, disrupting, coping, and healing/repairing harm (Hatchimonji et al., 2022; Poole et al., in press). The broader line of research, of which this intervention is a part, is informed by Harrell's (2000) model describing the links between racism‐related stress and mental health and Saleem and Byrd's (2021) model of racial socialization in schools. Our Actions Against Racism team has piloted educator trainings that promote educators' knowledge and skills to engage in actions against racism amongst staff and in interactions with students (Stoffers et al., 2024). School leaders and school staff are the primary practitioner partners for this project.

## ACTIONS AGAINST RACISM INTERVENTION DESCRIPTION

Actions Against Racism educator trainings are implemented school‐wide to promote educator confidence, attitudes, and skills for culturally affirming and race‐conscious practices. In our pilot school, the trainings were held monthly for 75–90 min. Each session connects to a specific action against racism: talking about racism, recognizing racism, disrupting racism, coping with racism, and healing from and repairing harm from racism. The skills targeted in each session draw from racial socialization (Stevenson, 2014) and racial healing (Menakem, 2021) literature to support recognizing and disrupting racism. Social‐emotional competencies to support coping and healing also draw from the racial socialization literature and also from evidence‐based social‐emotional learning (e.g., Gimbert et al., 2023) and trauma‐focused interventions (e.g., Herrenkohl et al., 2019). The theory of change of this intervention is that participating in the Actions Against Racism trainings leads to increased multicultural attitudes and efficacy, ultimately leading educators to engage in more culturally affirming and race‐conscious practices, which in turn supports students' racial coping, school belonging, and mental health. We found promising potential for these trainings to increase educators' attitudes and efficacy related to multiculturalism (Stoffers et al., 2024).

## AMBASSADORS IN ACTIONS AGAINST RACISM

We used Research‐Practice Ambassadors to learn: (1) how Actions Against Racism might fit in a new school context, (2) how Actions Against Racism implementation was experienced by educators in its first year, and (3) what problems of practice educators saw as relevant to Actions Against Racism. In each of these examples, we recruited Ambassadors who were passionate about social justice and curious about how to support school systems in combating racism. In most cases, Ambassadors' sociocultural identities reflected those of the majority of students in the partner school. Below, we describe examples of the three key Ambassador activities: (1) sharing strengths‐based observations, (2) cultivating authentic and equitable relationships, and (3) developing collaborative capstone projects. Table 2 provides excerpts from Ambassador notes that illustrate the observations of Ambassadors in each setting.

### Case examples: Strengths‐based observations

Working toward social justice requires disrupting deficit perspectives by valuing and uplifting strengths (Vetter et al., 2022). Further, by identifying and describing specific strengths and existing social justice‐oriented efforts or interactions, Ambassadors help the research team understand what individual‐, classroom‐, and school‐level practices already support equitable practices and the wellbeing of students, families, and staff. These strengths can then be a starting point for developing research questions and intervention ideas. Ambassadors share back their strengths‐based observations in field notes with the research team and in oral feedback and/or summary reports with the school community. Thus, at all stages of the research process, the Ambassador supports the bidirectional flow of information between researchers and practitioners. During their visits, Ambassadors complete structured observation forms developed by research and practice partners (Table 1, an example). Including Ambassadors, researchers, and practitioners in this process of creating the form is one way this process supports equitable partnerships with shared voice and power across the research and practice teams.

**Table 1:** Sample research practice ambassador observation form.

Visit Date:	Visit Time:
*Visit Overview/Description:*	
*Description of an interaction you observed:*	
*If relevant, please note what you observed in these categories:*	
1. *What cultures or identities were represented in the curriculum?*	
	*Any opportunities for growth?*
2. *What posters, books, or other materials demonstrated a priority of racial equity?*	
	*Any opportunities for growth?*
3. *How did teachers or school staff encourage students to bring their personal or emotional lives into the classroom? (Examples: Social‐emotional skill development, using “teachable moments” for SEL or conflict resolution)*	
	*Any opportunities for growth?*
4. How did you see teachers or school staff leaning in to courageous conversations? What was the topic?	
	*Any opportunities for growth?*

### School 1: Strengths‐based observations to cultivate relationships

We leveraged strengths‐based observations in a new RPP in a single K‐8 dual language school serving predominantly Latine students. The RPP Ambassador role served to strengthen relationships and understand current practices. At this point in the partnership process, the research team had no specific funded project or agenda, which provided flexibility to learn broadly about the social justice and equity context of this setting. In collaboration with the practitioner leaders in the school, we decided to embed one Ambassador in the school to observe classroom and school practices that promote inclusion and belonging. As the notes excerpts show (Table 2), the Ambassador in this setting observed the environmental and social‐emotional characteristics of the school and developed an interest in the school's approach to discipline.

**Table 2:** Excerpts from ambassador notes.

Partnership site	Note excerpts
School 1	“In honor of Black History Month, there were several posters in the hallways about Black culture, history, and beauty.”
	“[School staff] gave students a comfortable environment to talk about their feelings without invalidating any feelings. There was a student who came to her about an issue she was having in her relationship and [school staff] gave advice on how to combat the problem the student was having.”
School 2	“Many of [the counselor's] students have dealt with grief and have big feelings and she does an amazing job supporting her students. I am curious if the overarching theme of grief is common across Delaware schools, especially after the pandemic.”
	“We […] spoke on biases teachers hold and the ability for teachers to learn and grow. Her opinion was that people who want to grow will use tools like PDs to the best of their abilities while others will move through the motions of completing the mandatory work without actually learning anything.”
School 3	“Most kindergarten classrooms have “cool down” areas for students to collect themselves if they are feeling overwhelmed. Teachers can place them there or they can go themselves.”
	“Today, a student conducted the morning meeting. This made the student more engaged herself and her fellow students were excited to participate as well.”

As a participant‐observer, the Ambassador's strengths‐based reflections demonstrated the research team's dedication to getting to know the school community, thereby fostering trust as a foundation for collaborative projects. Because of the Ambassador's year‐long observations of strengths, the partnership was able to have a shared understanding of the school's approach to promoting positive discipline. Ultimately, the relationships built through the Ambassador role in School 1 served as a foundation to further collaborate on developing and piloting interventions to promote diversity, equity, inclusion, and belonging. In these formative stages of the partnership, the Ambassadors' involvement helped the research team understand how school values were enacted in daily practice. As this partnership continued to evolve, the Ambassadors' strengths‐based approach equipped the research team with knowledge of the social context, which supported the development of a collective vision and action plan.

### School 2: Strengths‐based observations to support bidirectional communication

We used RPP Ambassadors to support bidirectional communication during pilot development and implementation of the Actions Against Racism intervention in a single K‐8 school serving primarily Black and Brown students. The intervention development process included monthly meetings with a taskforce of seven educators (e.g., teachers, counselors, paraprofessionals) from the school. Two Ambassadors visited taskforce members approximately twice per week in the last 3 months of the pilot implementation school year. In this short time, the Ambassadors visited the classrooms of multiple educators, including teachers, paraprofessionals, and counselors who worked across grade levels. Ambassadors were able to observe the level of penetration of the educator‐focused intervention into the school setting and identify strengths in individuals' daily practice. The Ambassador note excerpts (Table 2) illustrate how one Ambassador observed the social‐emotional and social justice context of the school through the lens of the school counselor. Observations like these helped the research team understand the variability in penetration of the Actions Against Racism trainings beyond what was clear from the survey and focus group data collected by the research team. These observations gave voice to the daily lived experience of the practitioners putting the Actions Against Racism training into practice. Because of the Ambassador's observations, the research team had a clearer sense of the barriers to implementation faced by the educators in this school, such as demands on their time related to pressure to raise test scores. The research team was then equipped to share the concerns about time and prioritization with practitioners and school leaders, which set the stage for problem‐solving and planning. This involvement of the Ambassadors supported the research and practice teams in developing a shared understanding of the challenging expectations for educators in this school, which informed the next steps for the partnership.

### Case example: Cultivating relationships and developing a collaborative capstone

Once Ambassadors have become a trusted member of a school community, Ambassadors often engage in a capstone project in collaboration with specific practitioners. It is clear that an Ambassador has become a trusted member of the community when they start to experience more of the “participant” role during their school interactions. Typically, the problem of practice selected by the Ambassador results from months of dialog and relationship‐building with practitioners and leaders in the school community. To further build relationships between the research and practice teams, it is important that the capstone results in a product that is useful to the school community and especially to those individuals with whom they have formed authentic relationships. These capstone projects solidify the Ambassador's learning and provide an actionable guide, recommendations, or strategies from which students, educators, or families will benefit. Frequently, these projects are a joint effort between the Ambassador and the school staff or students with whom they have felt the deepest connection. We do not place a restriction on the topics of the capstone project because it is most important that the school community finds value in the outcome. Previous project examples include: an emotion‐focused problem‐solving process for middle school students referred to In‐School Suspension; a guide for integrating culturally responsive social‐emotional learning in math class; and a blog post describing the trauma‐informed implementation of a calm‐down corner for elementary students.

### School 3: Developing capstone project and amplifying voices

School 3 is a single K‐5 elementary school in a district that has agreed to partner with the research team in the future with the support of grant funding. This school serves primarily Black and Brown students and has recently seen a dramatic increase in newcomer students from Spanish‐speaking countries. Our team has embedded one undergraduate Ambassador in this school, with the goal to expand to additional schools in the district. The Ambassador in this setting spent one school year observing different classrooms, with a semi‐structured observation form to focus on strengths and areas for growth related to social‐emotional, trauma‐informed, and racial equity practices. The Ambassador note excerpts (Table 2) illustrate specific observations around the implementation of specific universal classroom climate and management strategies. A strength noted by this Ambassador was the consistent use of these strategies across many of the classrooms she visited. Guided by her own interests and observations and the Assistant Principal's assessment of need, the capstone project for this Ambassador was to support one first‐year teacher in planning for and implementing a calm‐down corner.

To accomplish this capstone, first, the RPP Ambassador built a relationship with the classroom teacher by providing emotional (e.g., providing a safe space for sharing challenges) or practical (e.g., making photocopies, walking a student to the nurse) support. Over several weeks, the Ambassador built relationships with all students in this classroom through consistent presence and participation in classroom routines. As both participant and observer, this Ambassador began to uplift the voices of newcomer students and students with social, emotional, and behavioral challenges by sharing observations with the teacher, research team, and school administrators. Together, the Ambassador and the teacher brainstormed how to support the needs of the diverse learners in the classroom and build an inclusive environment. In regular meetings with the Assistant Principal, the Ambassador also amplified the needs of the first‐year teacher who was overwhelmed with the demands of classroom management. The capstone was beneficial to the school and classroom as the Ambassador helped celebrate the strengths and gains with implementing the calm‐down space. The Ambassador's consistent presence, focus on strengths, and persistence in achieving the capstone goals demonstrated the investment of the research team in supporting the school. The practical support provided to the overwhelmed first‐year teacher also helped the teacher and the school support their diverse learners more effectively through inclusive morning meetings and the new calm‐down space. Ultimately, the RPP Ambassador process in this school helped identify shared priorities for future partnership: supporting new teachers in implementing identity‐affirming calm down corners and supporting newcomer families and multilingual learners.

## STRUCTURAL IMPACT OF RP AMBASSADORS ON PARTNERSHIP PROCESSES

These case vignettes demonstrate the varied ways that we have leveraged the Ambassador activities to support relationships, trust, and communication (Figure 2). Over time, the increased bidirectional communication, amplification of practitioner voices, and strengthened trusting relationships have the potential to contribute to larger structural transformation. For example, we have been able to leverage our relationships to support financial decisions and administrative communication. In School 1, administrator support and funding for the Diversity, Equity, Inclusion, and Belonging team were in jeopardy. The Ambassador's involvement contributed to trusting relationships that strengthened the partnership's ability to problem solve and respond to this challenge. Ultimately, the team was given the same level of funding. In School 2, the partnership used Ambassador observations to partner with an educator taskforce from a strengths‐based perspective. Together, the partnership was able to shift communication strategies regarding the Actions Against Racism work, which strengthened many educators' commitment to the Actions Against Racism classroom‐based practices. Without Ambassador involvement, it is unlikely that the research team would have had the understanding of the social context and trusting relationships to support these changes.

Any real change in school systems is necessarily a social process that requires shifts in power and actions guided by shared values across partners (Teemant et al., 2021). Embedding Ambassadors in RPPs can help partnerships arrive at shared values and a collective vision for structural and equitable transformation. Ambassadors can help shed light on areas of strength and barriers to acting in accordance with the collective vision. Over time, Ambassadors can support partnerships in the process of naming, reflecting, and acting toward social justice and equity (Freire, 1994; Teemant et al., 2014).

## STRENGTHS OF THE RP AMBASSADOR ROLE

Although RPPs have the potential to advance social justice and equity in education research (Henrick et al., 2019), in practice, these partnerships face immense challenges, particularly in enacting socially just and equitable partnership processes (Levac et al., 2019; Vetter et al., 2022). We have illustrated how the Ambassador role can be used to support socially just and equitable partnership processes by focusing on: (1) trusting relationships, (2) bidirectional communication, and (3) amplification of practitioner voices.

Henrick et al. (2019) highlight building trust and cultivating relationships as one of the five dimensions of effective RPPs. Building trusting relationships requires valuing and prioritizing relationships over the research agenda (Coburn et al., 2013). Yet, for researchers and practitioners alike, time demands can make it difficult to invest time into the partnership relationships. The Ambassador role supports relationships because Ambassadors have the flexibility to focus on problems of practice that matter to practitioners but may be only somewhat related to the specific interests of the broader RPP. This flexibility and ability to prioritize practitioner interests over research agenda are a strength of this Ambassador role. Further, the Ambassadors have more flexibility in how they spend their time in the school, which allows them to support practitioners in ways that practitioners may not have the bandwidth to do on their own.

The oft‐cited gap between research and practice is likely due, in part, to an inability of information to flow freely and equitably between researchers and practitioners (Farley‐Ripple et al., 2018). When Ambassadors share their strengths‐based observations with the research and practice teams, this process supports the bidirectional flow of information by sharing practitioner perspectives with the researchers and providing research‐team reflections to the practitioners. This process allows partnerships to co‐develop more contextually relevant research questions and methods that will ultimately have better implementation and greater impact on social justice and equity.

The way we have used the Ambassador role in RPPs is unique, particularly because of the emphasis on building relationships that are driven by the practitioners. Most research projects, and even RPPs that purport to be equity‐focused, fail to include all community stakeholders in the process (Levac et al., 2019). We have specifically used the RPP Ambassadors to build relationships and uplift the experiences of practitioners in school communities. As Ambassadors work to become members of the school community, they support the partnership in incorporating practitioners' lived experiences into the partnership processes. The long‐term presence of Ambassadors supports practitioners who experience being valued, heard, and seen as their concerns are taken seriously and their strengths are celebrated.

## LESSONS LEARNED AND FUTURE DIRECTIONS

We acknowledge that the RPP Ambassador is not inherently a tool for socially just and equitable processes. Like any tool, the Ambassador role could replicate power hierarchies and maintain the status quo. It is not the Ambassador role itself that strengthens relationships and communication in partnership processes. It is how the research and practice teams leverage this role in the context of RPPs that has the potential to support equitable partnership processes. Our experiences have led us to several potential future directions that could strengthen the potential impact of this role.

## CONTENDING WITH POWER

We have asserted that the Ambassador process has the potential to serve as a lever in shifting power hierarchies in school‐based research. Yet, a limitation of this work is that we have not yet contended with *how* to critically examine power and measure shifts over time. Future directions of this work would benefit from a structured power analysis to assess and reflect on power in the RPP and, ultimately, measure the impact of the RP Ambassador on shifting power toward a model of shared governance and power. Several tools offer frameworks for critically examining power relations. For example, Gaventa's (2006) “powercube” model describes the interplay of three dimensions of power, including those dimensions that are invisible but influential (e.g., culture). Alternatively, the “Net‐Map” model offers an interview‐based methodology for understanding power in social networks and communities (Schiffer & Hauck, 2010). Both approaches offer a framework for making complex and implicit power dynamics more explicit, which can contribute to the shared vision that is foundational to equitable partnership processes (Teemant et al., 2021).

Using a process for structured analysis of power would support key elements of shared governance, including a shared research vision, agreements about equitable data ownership/use, and a grievance/repair process for partners. To this point, we have supported Ambassadors in navigating these tensions through supervision and training. Yet, a more formal power analysis, with an explicit shared goal of understanding and shifting any inequities in power, would provide more structure and intentionality in shifting these dynamics. For example, the powercube model could be used to structure observations and reflections from the Ambassadors about spaces that appear to be *open* or *closed* to practitioners' perspectives regarding the research agenda. Ambassadors could then leverage these observations to have conversations with practitioners about what would be needed to shift dynamics in these spaces. The Net‐Map interview could be used by Ambassadors to collect information about historical and aspirational perspectives on power in the RPP. As one example, questions could be targeted toward who currently holds power in making decisions around data use/ownership. Results could inform how to address grievances/harm through a restorative process that moves the partnership toward shared power. Overall, the potential for these power analysis frameworks lies in the ability of the partnership to learn from these reflections and iteratively shift toward more open and invited spaces for all school community members.

## INVOLVING THE BROADER SCHOOL COMMUNITY

As described here, the Ambassador focuses on practitioner experiences in schools. This means that, currently, student and family voices are not represented through this Ambassador process. But, this role can be used to uplift the student or family experience or any other stakeholder group. Similarly, while we have found the use of university students in this role to be advantageous, this also means that there are some inherent power imbalances that may be difficult to overcome. Thus, it may be even more impactful to have members from the local community in this Ambassador role. To move in this direction, the partnership would need to agree on a recruitment and funding strategy, giving full consideration to making the Ambassador position accessible to all community stakeholders represented in a school community. This would mean ensuring disability, cultural, and linguistic access for community‐hired Ambassadors. Disability access might include ensuring physical and virtual spaces are designed to promote access for all, and if there are limitations in these spaces, addressing them together as a partnership. Cultural access might include partnering with trusted community organizations to co‐design the Ambassador role and prioritize hiring Ambassadors who have cultural familiarity and shared histories with the families represented in the school. Linguistic access might include training research team members in linguistic justice to ensure Ambassadors are empowered to use their full linguistic repertoire in the school and in collecting observations and data. It may also be important to build interpretative services into the role so that Ambassadors can fully participate in decision‐making.

## FUNDING FOR THE AMBASSADOR ROLE

Decisions about how to fund the Ambassador role contribute to the complex power dynamics among the members of the RPP and the school community. Using university students, the RP Ambassador role can be efficient and inexpensive, which can be an important asset to developing a practitioner‐driven research agenda. Yet, there is still some cost associated with compensating the Ambassadors (through work study or course credit if not a research grant), and there is significant personnel time associated with supervising, training, and coordinating Ambassadors. We advise against Ambassadors as volunteers because of the need to compensate them for their contributions. A drawback to the university funding system is that relying on course credit or work study funding makes it harder to recruit Ambassadors who are from the local community. While it is possible to run this program with minimal external funding, it is difficult to sustain long‐term involvement without any funds from either a research grant or the partnering district. We have seen the Ambassador process most easily sustained when districts and academic centers partner in providing funding to support supervision and coordination of Ambassadors.

## CONCLUSION

Embedding the Research‐Practice Ambassador role in school‐based intervention research offers a sustainable way to promote equitable collaboration and communication across research and practice teams by supporting socially just and equitable partnership processes. We have argued that if the Research‐Practice Ambassador is thoughtfully used across the stages of the research process, then this model is suited to improve upon existing RPPs and promote authentic relationships and bidirectional communication, ultimately working towards improved, sustainable, collaborative partnership processes.

## AUTHOR CONTRIBUTIONS

Danielle Hatchimonji and Lauren McNeela conceptualized and drafted the manuscript. Melissa Stoffers, Tia Barnes, Mariam Berthe, Kira Branch, and Danielle Hatchimonji developed and participated in the methodology described here and provided feedback on the manuscript. Amanda Parks and Danika Perry support revisions and manuscript development. Zumana Noor created figures and supported manuscript development.

## CONFLICT OF INTEREST STATEMENT

The authors declare no conflicts of interest.

## ETHICS STATEMENT

This paper describes a methodology for supporting research practice partnerships without presenting any research data.

## Data Availability

There is no research data presented in this paper.
